# Virtual reality (VR) therapy for patients with psychosis: satisfaction and side effects

**DOI:** 10.1017/S0033291722001167

**Published:** 2023-07

**Authors:** Daniel Freeman, Laina Rosebrock, Felicity Waite, Bao Sheng Loe, Thomas Kabir, Ariane Petit, Robert Dudley, Kate Chapman, Anthony Morrison, Eileen O'Regan, Charlotte Aynsworth, Julia Jones, Elizabeth Murphy, Rosie Powling, Heather Peel, Harry Walker, Rory Byrne, Jason Freeman, Aitor Rovira, Ushma Galal, Ly-Mee Yu, David M. Clark, Sinéad Lambe

**Affiliations:** 1 Department of Psychiatry, University of Oxford, Oxford, UK; 2 Oxford Health NHS Foundation Trust, Oxford, UK; 3 The Psychometrics Centre, University of Cambridge, Cambridge, UK; 4 McPin Foundation, London, UK; 5 Cumbria, Northumberland, Tyne, and Wear NHS Foundation Trust, Newcastle upon Tyne, UK; 6 Newcastle University, Newcastle upon Tyne, UK; 7 Avon and Wiltshire Mental Health Partnership (AWP) NHS Trust, Bath, UK; 8 Greater Manchester Mental Health Foundation Trust, Manchester, UK; 9 Division of Psychology and Mental Health, University of Manchester, Manchester, UK; 10 Nottinghamshire Healthcare NHS Foundation Trust, Nottingham, UK; 11 Oxford Primary Care Clinical Trials Unit, Nuffield Department of Primary care Health Sciences, University of Oxford, Oxford, UK; 12 Department of Experimental Psychology, University of Oxford, Oxford, UK

**Keywords:** Psychosis, satisfaction, schizophrenia, side effects, virtual reality

## Abstract

**Background:**

Automated virtual reality therapies are being developed to increase access to psychological interventions. We assessed the experience with one such therapy of patients diagnosed with psychosis, including satisfaction, side effects, and positive experiences of access to the technology. We tested whether side effects affected therapy.

**Methods:**

In a clinical trial 122 patients diagnosed with psychosis completed baseline measures of psychiatric symptoms, received gameChange VR therapy, and then completed a satisfaction questionnaire, the Oxford-VR Side Effects Checklist, and outcome measures.

**Results:**

79 (65.8%) patients were very satisfied with VR therapy, 37 (30.8%) were mostly satisfied, 3 (2.5%) were indifferent/mildly dissatisfied, and 1 (0.8%) person was quite dissatisfied. The most common side effects were: difficulties concentrating because of thinking about what might be happening in the room (*n* = 17, 14.2%); lasting headache (*n* = 10, 8.3%); and the headset causing feelings of panic (*n* = 9, 7.4%). Side effects formed three factors: difficulties concentrating when wearing a headset, feelings of panic using VR, and worries following VR. The occurrence of side effects was not associated with number of VR sessions, therapy outcomes, or psychiatric symptoms. Difficulties concentrating in VR were associated with slightly lower satisfaction. VR therapy provision and engagement made patients feel: proud (*n* = 99, 81.8%); valued (*n* = 97, 80.2%); and optimistic (*n* = 96, 79.3%).

**Conclusions:**

Patients with psychosis were generally very positive towards the VR therapy, valued having the opportunity to try the technology, and experienced few adverse effects. Side effects did not significantly impact VR therapy. Patient experience of VR is likely to facilitate widespread adoption.

## Introduction

A common question regarding the use of virtual reality (VR) with patients with psychosis is whether there are adverse effects, such as patients becoming suspicious of the technology or finding it hard to distinguish between the virtual and real worlds. The issue of simulator sickness is also often raised in discussions of VR (Chang, Kim, & Yoo, 2020). In contrast, our experience delivering VR therapy is that patients with psychosis readily understand the technology when it is explained, are excited about trying VR, and seldom get suspicious when using it. However, there has been no systematic collection of data on VR side effects or adverse reactions for this patient group. There are data, however, showing that when VR tasks are appropriately designed, and suitable hardware used, patients with current psychotic experiences typically do not experience simulator sickness (Fornells-Ambrojo et al., 2008; Freeman, Pugh, Vorontsova, Antley, & Slater, 2010). Spurred by the arrival of consumer-focussed immersive equipment, the use of VR for mental health conditions is increasing. To enable the scaling up of effective psychological interventions, we have been developing automated psychological therapies within VR (e.g. Freeman et al. 2018, 2019a). A virtual coach guides the user through the sessions and therapeutic tasks are embedded within the VR scenarios. However, necessary (but not sufficient) conditions for the adoption of VR within mental health services will be user satisfaction with automated delivery and the minimisation of adverse treatment reactions. Therefore, we studied satisfaction, side effects, and also the potentially positive experiences of access to VR technology for patients with psychosis receiving the gameChange therapy.

gameChange is a six-session automated VR cognitive therapy targeting agoraphobic avoidance of everyday situations and distress when in those situations. Designed with people with lived experience, patients can evaluate their fears in six different scenarios: leaving the front door to step into the street, getting onto a bus, and visiting a café, a doctor's surgery, a shop, and a pub (Knight et al., 2021; Lambe et al., 2020). A virtual coach, built into the programme, provides instruction, information, and encouragement. Delivery of the VR intervention is supported by a peer support worker, assistant psychologist, or psychologist. Their role is to help set up the hardware, explain what is going to happen, and help organise homework tasks to consolidate the learning from VR. gameChange was evaluated in a randomised controlled trial with 346 patients with psychosis (Freeman et al., 2022). For the therapy delivery that was uninterrupted by the coronavirus pandemic, uptake was high, with 89% of patients receiving at least three VR sessions (predefined as a dose). Compared to treatment as usual, the VR therapy led to significant end of treatment reductions (Cohen's *d* = 0.2) in agoraphobic avoidance and distress. However, there was moderation of treatment effects. Patients with severe agoraphobic avoidance showed the greatest benefits, with large effect size improvements (Cohen's *d* = 0.8) at the six-month follow-up. This was a quarter of the patient population in the trial. There was little evidence of benefit for patients with low levels of agoraphobia. Systematic blinded inspection of medical notes showed that levels of serious adverse events (e.g. suicide attempts, hospital admission), and adverse events close to being serious (e.g. self-injury not requiring treatment), were comparable in the VR and control arms of the trial. There were no serious adverse events considered related to the VR therapy. This is consistent with clinical trials for other conditions, in which no serious adverse events attributable to use of VR have been detected (e.g. Beidel et al., 2019; Donker et al., 2019; Spiegel et al., 2019).

At their last gameChange VR therapy session, patients were asked to complete assessments of satisfaction and VR side effects. Side effects were defined as unwanted negative events that patients attributed to use of VR (hardware or software). They comprised potentially milder subjective experiences (e.g. eye strain, feelings of panic, increase in voices or paranoia) compared to the serious adverse events, defined by medical device trial guidelines (e.g. death, life-threatening injury, hospitalisation), that were scrutinised by formal trial procedures and presented in the trial primary report. We set out to assess: the degree of satisfaction with automated VR therapy; the occurrence of a range of side effects and how they may cluster into factors; whether side effects might affect therapy uptake, outcomes, and satisfaction; whether individual psychiatric symptoms assessed before VR therapy predicted the occurrence of side effects; and how patients felt about being given access to VR technology. In this paper, we report on these data for the first time.

## Method

The gameChange trial received approval from an NHS Research Ethics Committee (NHS South Central-Oxford B Research Ethics Committee, ref 19/SC/0075), was registered prospectively (ISRCTN17308399), and the protocol published at the start of the trial (Freeman et al., 2019b). Patients completed baseline measures before potentially receiving VR therapy. The main trial outcome measure, the Oxford Agoraphobic Avoidance Scale (O-AS) (Lambe et al., 2021), was repeated at 6 (end of treatment) and 26 weeks after randomisation.

### Participants

The main inclusion criteria for patients in the gameChange trial were: adults aged 16 years or older; attending an NHS mental health trust for the treatment of psychosis; clinical diagnosis of schizophrenia spectrum psychosis (F20-29) or an affective diagnosis with psychotic symptoms (F31.2, 31.5, 32.3, 33.3) (ICD-10) (WHO, 2004); and having self-reported difficulties going outside the home primarily due to anxiety that they would like treated. The main exclusion criteria were: photosensitive epilepsy; significant visual, auditory, or balance impairment; in forensic settings or Psychiatric Intensive Care Unit (PICU); organic syndrome; primary diagnosis of alcohol or substance disorder or personality disorder; or current active suicidal plans.

122 participants who attended at least one gameChange therapy session provided data for the current study. 118 patients completed both the satisfaction questionnaire and side effects questionnaire. One person completed the satisfaction questionnaire only and three people completed the side effects questionnaire only. These participants were drawn from the 174 patients randomised to receive the gameChange therapy in the trial. 13 patients attended no VR sessions, with eight of these individuals prevented from doing so because of COVID-19 restrictions. The current report has therefore been compiled from the responses of 122 (75.8%) patients from the 161 who tried VR. It is of note that for 27 patients who were randomised immediately before COVID-19 lockdowns began, provision of VR therapy and data collection were adversely affected by NHS trust pandemic restrictions on face-to-face contact.

The 122 participants in the current study did not differ at baseline from the other 52 patients randomised to therapy in gender, chi-square (*df* = 1) = 0.209, *p* = 0.647, age, *t*(*df* = 172) = −0.851, *p* = 0.396, antipsychotic medication dose, *t*(*df* = 159) = −0.626, *p* = 0.532, anxious avoidance (O-AS), *t*(*df* = 171) = 0.951, *p−*0.343, anxious distress (O-AS), *t*(*df* = 172) = −0.610, *p* = 0.543, paranoia (R-GPTS total), *t*(*df* = 152) = 0.697, *p* = 0.487, threat cognitions (O-CDQ), *t*(*df* = 169) = 0.272, *p* = 0.786, anxious avoidance (O-CDQ), *t*(*df* = 169) = −0.720, *p* = 0.472, within-situation defence behaviours (O-CDQ), *t*(*df* = 169) = 1.178, *p* = 0.241, occurrence of negative voices when outside, *t*(*df* = 152) = 0.210, *p* = 0.834, or hopelessness (BHS), *t*(*df* = 152) = 1.607, *p* = 0.110. The participants in this study did attend a greater number of VR sessions (mean = 6.13, s.d. = 1.30) than those who did not take part (mean = 2.52, s.d. = 2.09), *t*(*df* = 72.09) = −11.703, *p* < 0.001.

### Assessments

*Client Satisfaction Questionnaire* (CSQ) (Attkisson & Greenfield, 1999; Attkisson & Zwick, 1982). Four items were used from the CSQ, with two additional questions about the number of sessions and experience of the staff member supporting VR delivery (see Table 1 for all questionnaire items). The Cronbach's *α* for the six items in this study was 0.58, and items are reported individually.

**Table 1.** Satisfaction with gameChange VR therapy

How would you rate the quality of the virtual reality therapy you have received? *n* (%)				
	Excellent	Good	Fair	Poor
	77 (64.2%)	40 (33.3%)	3 (2.5%)	0
To what extent has virtual reality therapy helped you feel more confident when outside in everyday situations? *n* (%)				
	Yes, it helped a great deal	Yes, it helped a bit	No, it didn't really help	No, it seemed to make things worse
	61 (50.8%)	53 (44.2%)	5 (4.2%)	1 (0.8%)
What did you think of the number of virtual reality therapy sessions that you received? *n* (%)				
		Too many sessions	Just the right number of sessions	Too few sessions
		1 (0.8%)	94 (78.3%)	25 (20.8%)
Was the member of staff supporting you with the VR therapy helpful? *n* (%)				
	Yes, they were very helpful	Yes, they were somewhat helpful	No, they didn't really help	No, they were very unhelpful
	118 (98.3%)	2 (1.7%)	0	0
How likely are you to recommend virtual reality therapy to friends and family if they needed similar care or treatment? *n* (%)				
Extremely likely	Likely	Neither likely or unlikely	Unlikely	Extremely unlikely
63 (52.5%)	46 (38.3%)	10 (8.3%)	1 (0.8%)	0
In an overall, general sense, how satisfied are you with the virtual reality therapy you have received? *n* (%)				
	Very satisfied	Mostly satisfied	Indifferent or mildly dissatisfied	Quite dissatisfied
	79 (65.8%)	37 (30.8%)	3 (2.5%)	1 (0.8%)

*Oxford – VR Side Effects Checklist* (*O-VRSE*). A long list of items was generated by the University of Oxford clinical psychologists, experienced in VR therapy design and delivery, concerning the potential physical and psychological effects of wearing a VR headset; the psychological experience of the simulations; the potential physical and psychological effects after being in VR, including feeling of sickness, occurrence of psychotic symptoms, and difficulties distinguishing between real and virtual worlds; and potential positive experiences of having access to VR therapy. The initial item list was added to, and item wording improved, in meetings with the gameChange Lived Experience Advisory Panel (LEAP) comprising ten people with lived experience of psychosis who had also tried VR. The final item pool comprised 33 items. Participants are asked to report whether they had had any of the experiences listed during the provision of VR therapy (Yes or No) (see Table 2 for all items). The questions were designed to have content that could be answered with a dichotomous response (e.g. ‘While I was wearing the headset, I fell and injured myself.’). We were interested in clear, reportable occurrences of side effects. No was coded as 0, and Yes was coded as 1. Higher scores indicate a greater rate of endorsement.

**Table 2.** Endorsement of Oxford-VR side effects checklist (O-VRES) items (ranked by frequency)

Negative items	No (*n*)	Yes (*n*)
I couldn't concentrate on my session because I was constantly thinking about what else might be happening in the room.	103	17
	85.8%	14.2%
Using the headset gave me a lasting headache.	111	10
	91.7%	8.3%
The headset made me feel trapped/claustrophobic and I had a panic attack.	112	9
	92.6%	7.4%
I couldn't fully engage in the session because I was constantly thinking about crashing into something.	113	8
	93.4%	6.6%
Using the headset strained my eyes so I couldn't see properly.	113	8
	93.4%	6.6%
In the days after using VR, I couldn't tell the difference between the computer VR world and the real world.	115	6
	95.0%	5.0%
As a result of using VR, I was really confused about what was real and what was not real.	115	6
	95.0%	5.0%
After my session, I was really concerned that the headset had messed with my thoughts.	115	6
	95.0%	5.0%
Wearing the headset made my voices worse for the rest of the day. (not applicable for 20 patients)	96	5
	95.0%	5.0%
While using VR, I felt so sick that I had to stop.	115	5
	95.8%	4.2%
The people in VR were so creepy that I did not want to continue with the therapy.	115	5
	95.8%	4.2%
Going into the VR environments made me even more worried about other people.	115	5
	95.8%	4.2%
Wearing the headset caused me pain and discomfort for quite some time after the session had finished.	116	5
	95.9%	4.1%
After using VR, the everyday world felt very unreal.	116	5
	95.9%	4.1%
After using VR, I felt very disconnected from the real world.	116	5
	95.9%	4.1%
After wearing the headset, I felt so unsteady that I had difficulties walking.	118	3
	97.5%	2.5%
After my session, I got worried and fearful about what the headset had done to me.	118	3
	97.5%	2.5%
For hours after using VR, I felt sick/unwell.	118	3
	97.5%	2.5%
While I was wearing the headset, I walked into something and injured myself.	119	2
	98.3%	1.7%
Wearing the headset left me with worrying/distressing marks on my face for quite some time.	119	2
	98.3%	1.7%
The virtual coach was very unhelpful and put me off the therapy.	119	2
	98.3%	1.7%
I couldn't concentrate on my session because I was constantly thinking about what the headset might be doing to me.	119	1
	99.2%	0.8%
Going into the VR environments made me have panic attacks.	120	1
	99.2%	0.8%
The therapy got too hard, too quickly, and I felt defeated.	120	1
	99.2%	0.8%
While I was wearing the headset, I fell and injured myself.	121	0
	100%	0%
VR made me throw up.	121	0
	100%	0%
After using VR, I began to see disturbing things that other people couldn't see.	121	0
	100%	0%
Positive items		
I felt proud of myself for being able to use the VR.	22	99
	18.2%	81.8%
Receiving this new and high-tech therapy made me feel valued.	24	97
	19.8%	80.2%
Using the VR equipment made me feel optimistic.	25	96
	20.7%	79.3%
The headset felt comfortable	26	95
	21.5%	78.5%
Using the VR equipment made me feel excited.	45	76
	37.2%	62.8%
Using the VR equipment made me feel really special.	80	40
	66.7%	33.3%

*Oxford Agoraphobic Avoidance Scale* (O-AS) (Lambe et al., 2021). The O-AS lists eight simple tasks progressing in difficulty from ‘Stand outside your home on your own for 5 min’ through ‘Travel on your own on the bus for several stops’ to ‘Sit in a café on your own for 10 min’. Participants are asked whether they could do the task now or whether they could not because of anxiety (Yes = 0, No = 1), which provides the avoidance score (0–8). For each task participants are also asked on a 0 (no distress) to 10 (extreme distress) scale how anxious they would feel doing it. These distress scores are summed to provide an overall distress score. Higher scores indicate greater agoraphobic symptoms. The O-AS was the main outcome measure in the gameChange trial.

*Revised Green et al. Paranoid Thoughts Scale* (R-GPTS) (Freeman et al., 2021). The R-GPTS comprises an eight-item ideas of reference scale and a 10-item ideas of persecution scale. Each item is rated for the past fortnight on a 5-point (0 to 4) scale. Higher scores indicate greater levels of paranoia.

*Mobility Inventory for Agoraphobia – Alone subscale* (MIA) (Chambless, Caputo, Jasin, Gracely, & Williams, 1985). The degree of current avoidance because of anxiety is rated on a 1 (never avoid) to 5 (always avoid) scale for each of 26 situations (e.g. supermarkets, riding in buses, lifts). Higher scores indicate greater anxious avoidance.

*Paranoia Worries Questionnaire* (Freeman et al., 2020). This five-item questionnaire assesses the degree to which an individual has been worrying in the past month about others trying to harm them. Each item is rated on a 0 (none of the time) to 4 (all of the time) scale. Higher scores indicate higher levels of worry with a paranoia content.

*Patient Health Questionnaire* (PHQ-9) (Kroenke, Spitzer, & Williams, 2001). This scale assesses depressive symptoms over the past fortnight. Each of the nine items is rated on a 0 (not at all) to 3 (nearly every day) scale. Higher scores indicate higher levels of depression.

*Questionnaire about the Process of Recovery* (QPR) (Law, Neil, Dunn, & Morrison, 2014; Neil et al., 2009). This is a 15-item questionnaire developed collaboratively by service-user researchers and clinicians assessing recovery. Items are rated on a five-point scale from 0 (strongly disagree) to 4 (strongly agree). Higher scores indicate greater recovery.

*Beck Hopelessness Scale* (BHS) (Beck & Steer, 1988). Twenty statements about feelings of hopelessness in the past week are rated as True or False. Higher scores reflect greater hopelessness.

*Oxford Cognitions and Defences Questionnaire* (O-CDQ) (Rosebrock et al., 2022). The O-CDQ comprises three subscales assessing threat cognitions that may contribute to agoraphobia (14 items), anxious avoidance (11 items), and within-situation defence behaviours (8 items). Each item is rated on a scale from 0 (never) to 3 (always). Higher scores on each subscale indicate higher levels of the anxiety-related psychological factor.

*Negative Voices When Outside*. Five items assessed auditory hallucinations that inhibit a person from participation in everyday social situations (e.g. ‘I hear voices that make it difficult to go outside’, ‘As soon as I start thinking about going out, my voices tell me bad things are going to happen’). Each item is rated on a 0 (not at all) to 4 (daily) scale. The Cronbach's *α* for this scale is 0.93. Higher scores indicate greater occurrence of voices.

### Intervention

The gameChange VR therapy is a VR application recommended for adults (16+) who are anxious about everyday social situations. The software is intended to reduce anxiety around other people. The treatment is a CE marked Class I Active Medical Device- Z301 (Standalone Software), in conformity with the essential requirements and provisions of the EC Directive 93/42/EEC (Medical Devices). The hardware used in the trial was an HTC Vive Pro headset and Dell G5 15 5590 laptop with Intel i7 CPU, 16GB of RAM memory, and Nvidia GeForce RTX2060 graphics card. The treatment was designed to be delivered in approximately 6 sessions, each involving thirty minutes in VR. A mental health worker – peer support worker, assistant psychologist, or clinical psychologist – was in the room when the treatment was provided. The staff member set up the hardware and helped the person make the most of the learning from the programme. The gameChange VR therapy is a cognitive treatment that aims for patients to relearn safety by testing their fear expectations around other people. The VR therapy participant typically stands and can walk a few paces in the scenarios. Within the VR environments a virtual coach guides the person through the treatment. The coach encourages the dropping of defence (safety-seeking) behaviours, the evaluation of fears, and elicits feedback to tailor the progression of the treatment. When first entering VR, the patient goes into the coach's virtual office and is guided in how to use VR. At the beginning of the first session, the virtual coach explains the rationale behind the treatment, and the participant selects one of six VR scenarios. Each scenario comprises five levels of difficulty and participants work their way through the tasks in each level. The participant can choose a different scenario in each session or repeat a previous scenario. A full description of the design process and VR therapy is provided in two separate publications (Knight et al., 2021; Lambe et al., 2020).

### Analysis

All analyses were conducted in SPSS Version 27.0 (IBM, 2020) or the R programming language (R Core Team, 2021). The psych R package (Revelle, 2021) was used to perform the exploratory factor analysis (EFA) and related tests. Descriptive statistics were used to present the satisfaction ratings and the prevalence of side effects.

For the Oxford-VR Side Effects Checklist the Kaiser Meyer Olkin (KMO) sampling of adequacy and Bartlett's test of sphericity were used to evaluate whether the scale was suitable for conducting factor analysis (Hair, Black, Babin, Anderson, & Tatham, 2006; Kaiser, 1970, 1974). Items with a KMO value of less than 0.5 were considered unacceptable and removed from further analysis (Hair et al., 2006). A statistically significant Bartlett's test of less than 0.05 indicates that sufficient correlations exist between the items in the scale to conduct factor analysis (Hair et al., 2006). Next, a parallel analysis (PA) was conducted using the tetrachoric correlation matrix to determine the number of dimensions to retain (Velicer, Eaton, & Fava, 2000). The rationale of PA is that nontrivial components from actual data with a valid underlying factorial structure should have eigenvalues larger than parallel components derived from random simulated data of equal sample size and having the same number of variables (Lautenschlager, 1989). Only the factors that corresponded to actual eigenvalues greater than the parallel average random eigenvalues were retained. Moreover, the Kaiser greater than 1 criterion (K1) was utilised to keep factors with eigenvalues greater than 1 (Kaiser, 1960). Subsequently, EFA based on the retained dimensions was performed to evaluate the factorial structure of the scale. Oblimin rotation was applied and the minimal residual approach for the factoring method was used. Items with factor loadings of less than 0.4 and multiple cross-loadings were removed. The factor scores were estimated using the least-squares regression approach (Thurstone, 1935), which is a procedure that maximises the validity of the estimates and is most suitable for use as predictors in subsequent regression analyses (DiStefano, Zhu, & Mindrila, 2009; Grice, 2001). As estimated factor scores are not uniquely defined, different EFA selection methods are likely to change these scores, which could affect subsequent statistical modelling and interpretation if they differed widely (Beauducel, 2005; Grice, 2001; Zuccaro, 2010). Therefore, the validity of the factor solutions was evaluated based on three criteria: evidence of moderate to strong correlational relationships between the estimated factor scores and raw scores of the factors; the extent to which the factors were sufficiently unique/related to the other factors in the same analysis; and correlational accuracy (the extent to which the correlational relationship of the estimated factor scores were similar to the correlations of the factors themselves). Using correlational analysis, the factor scores derived from EFA were then used to establish the relationship with the other assessments. The analyses were done at a pair-wise level to retain as many participants as possible.

## Results

The basic demographic and clinical details of the participant group are summarised in Table 3.

**Table 3.** Demographic and clinical information about the participants

	VR therapy group (*N* = 122)
Age (years)	
Mean (s.d.)	37.3 (13.2)
Gender, *n* (%)	
Female	39 (32.0%)
Male	83 (68.0%)
Other	0
Prefer not to say	0
Marital status, *n* (%)	
Single	91 (75.8%)
Married/civil partnership	14 (11.5%)
Cohabiting	5 (4.2%)
Separated	2 (1.7%)
Divorced	7 (5.8%)
Widowed	1 (0.8%)
Ethnicity, *n* (%)	
White	105 (86.1%)
Black British	1 (0.8%)
Black African	1 (0.8%)
Black Caribbean	0
Indian	0
Black Other	1 (0.8%)
Pakistani	2 (1.6%)
Other	12 (9.8%)
Service type, *n* (%)	
Community mental health team	72 (59.0%)
Early intervention service	46 (37.7%)
In-patient	4 (3.3%)
Employment, *n* (%)	
Employed full-time (paid)	8 (7.3%)
Employed part-time (paid)	3 (2.8%)
Employed full-time (voluntary)	0
Employed part-time (voluntary)	1 (0.9%)
Unemployed (on benefits)	76 (69.7%)
Unemployed (not on benefits)	7 (6.4%)
Student or in training full-time	3 (2.8%)
Student or in training part-time	2 (1.8%)
Self-employed	4 (3.7%)
Home-maker	0
Carer	1 (0.9%)
Retired	4 (3.7%)
Missing	13
Mental health diagnosis, *n* (%)	
Schizophrenia	52 (42.6%)
Schizotypal disorder	1 (0.8%)
Delusional disorder	2 (1.6%)
Brief psychotic disorders	3 (2.5%)
Schizoaffective disorder	12 (9.8%)
Other psychotic disorder	2 (1.6%)
Unspecified psychosis	40 (32.8%)
Bipolar disorder with psychotic features	2 (1.6%)
Depressive disorders with psychotic features	5 (4.1%)
Major depressive disorder with psychotic features	3 (2.5%)
Antipsychotic medication	
Yes, *n* (%)	107 (89.2%)
No, *n* (%)	9 (10.8%)
Missing, *n* (%)	1
Chlorpromazine equivalent dose mean (s.d.)	378.7 (320.2)

Levels of patient satisfaction with gameChange VR therapy, which were very high, are summarised in Table 1. Almost all the patients rated the quality of the VR therapy as good or excellent, and reported that it helped with their anxiety, that the staff member was supportive, that they would recommend the VR therapy to others, and that they were satisfied. About one-fifth of patients would have liked additional VR sessions. A small number of patients were dissatisfied with the VR therapy.

The occurrence of each side effect, ranked by frequency, is displayed in Table 2. The most common side effect (‘I couldn't concentrate on my session because I was constantly thinking about what else might be happening in the room’) occurred for 17 (14.2%) patients. The mean number of side effects endorsed (out of a possible score of 27) was 2.45 (s.d. = 3.9) (median = 1). In contrast the mean number of positive items endorsed (out of a possible score of 6) was 4.15 (s.d. = 1.6) (median = 4). The most common positive item (‘I felt proud of myself for being able to use the VR’) was endorsed by 99 (81.8%) patients.

118 patients completed all Oxford-VR Side Effects Checklist items and their data were used in the factor analysis. Three items from the Oxford-VR Side Effects Checklist (‘While I was wearing the headset, I fell and injured myself’, ‘VR made me throw up’, ‘After using VR, I began to see disturbing things that other people couldn't see’) were excluded from further analysis because all participants answered ‘No’ to these items. Six items (‘Wearing the headset left me with worrying/distressing marks on my face for quite some time’, ‘Using the headset strained my eyes so I couldn't see properly’, ‘Wearing the headset made my voices worse for the rest of the day’, ‘Going into the VR environments made me even more worried about other people’, ‘The virtual coach was very unhelpful and put me off the therapy’, ‘The therapy got too hard, too quickly, and I felt defeated’) had a KMO value less than 0.5 and were removed from the analysis. The final set of items (*n* = 24) had an overall KMO of 0.65 with a Bartlett's test of sphericity of *p* < 0.0001, indicating that factor analysis could be conducted with the data. According to a PA the parallel average random simulated eigenvalue was 0.28, and the top seven actual eigenvalues were 8.22, 2.62, 1.78, 1.22, 0.88, 0.69, and 0.26, respectively. The result indicated the recovery of six factors, given that the last actual eigenvalue (0.26) was lower than the parallel average random simulated eigenvalue (0.28). Furthermore, only the first four factors had eigenvalues greater than 1. Given that the difference between the fourth (1.22) and fifth (0.88) eigenvalue was considered substantial, only the top four factors were retained. An initial EFA of 24 items identified four distinct factors: F1: Worries following VR; F2: Feelings of panic using VR; F3: Feeling valued; F4: Difficulties concentrating when wearing a headset. Two items (‘For hours after using VR, I felt sick/unwell’, ‘The headset felt comfortable’) with multiple cross-loadings of over 0.35 were deleted to obtain a cleaner factor structure. EFA of the remaining 22 items supported a four-factor structure which explained 66% of the variance, and all items had factor loading greater than 0.4 (see Table 4). Correlations between the factors are summarised in Supplementary material (online Supplementary Tables S1 and S2).

**Table 4.** Final items and loadings from exploratory factor analysis

Items	F1: Worries following VR	F2: Feelings of panic using VR	F3: Feeling valued	F4: Difficulties concentrating when wearing a headset
I couldn't concentrate on my session because I was constantly thinking about what the headset might be doing to me.	0.77			
After wearing the headset, I felt so unsteady that I had difficulties walking.	0.48			
After my session, I was really concerned that the headset had messed with my thoughts.	0.76			
After my session, I got worried and fearful about what the headset had done to me.	0.89			
In the days after using VR, I couldn't tell the difference between the computer VR world and the real world.	0.78			
As a result of using VR, I was really confused about what was real and what was not real.	0.65			
After using VR, the everyday world felt very unreal.	0.82			
After using VR, I felt very disconnected from the real world.	0.76			
The headset made me feel trapped/claustrophobic and I had a panic attack.		0.62		
Wearing the headset caused me pain and discomfort for quite some time after the session had finished.		0.66		
Using the headset gave me a lasting headache.		0.51		
While using VR, I felt so sick that I had to stop.		0.89		
The people in VR were so creepy that I did not want to continue with the therapy.		0.80		
Going into the VR environments made me have panic attacks.		0.86		
Using the VR equipment made me feel really special.			0.67	
I felt proud of myself for being able to use the VR.			0.71	
Receiving this new and high-tech therapy made me feel valued.			0.86	
Using the VR equipment made me feel excited.			0.56	
Using the VR equipment made me feel optimistic.			0.64	
While I was wearing the headset, I walked into something and injured myself.				0.41
I couldn't concentrate on my session because I was constantly thinking about what else might be happening in the room.				0.99
I couldn't fully engage in the session because I was constantly thinking about crashing into something.				0.69

The validity of the factor scores was evaluated on the three criteria. Strong correlational relationships were observed between the estimated factor scores and raw scores of the four factors (F1: Worries following VR, F2: Feelings of panic using VR, F3: Feeling valued, and F4: Difficulties concentrating when wearing a headset; *r* = 0.80, 0.75, 0.92, 0.87, respectively). The small to moderate factor correlations based on the factors and estimated factor scores indicated the presence of distinct factors (Supplementary materials online Supplementary Tables S1 and S2). Finally, the direction of the correlational relationships is similar for factor and estimated factor scores (as seen in online Supplementary Tables S1 and S2), although the correlations are moderately inflated for the estimated factor scores. The results suggest that the estimated factor scores adequately represent the factors.

Age was not associated with difficulties concentrating when wearing a headset, *r* = −0.15, *p* = 0.111, *n* = 118, feelings of panic using VR, *r* = −0.11, *p* = 0.229, *n* = 118, worries following VR, *r* = −0.07, *p* = 0.450, *n* = 118, or feeling valued because of VR provision, *r* = 0.11, *p* = 0.220, *n* = 118. O-VRSE factor scores did not differ between males and females (all *p* values > 0.1). Difficulties concentrating when wearing a headset did not differ if the patient was seen at home or in the clinic, *t*(*df* = 102.1) = 0.655, *p* = 0.499. Associations of the O-VRSE factor scores with baseline symptoms, therapy time, and trial primary outcomes are summarised in Table 5. There were no associations of baseline standard symptom scores (e.g. paranoia, voices, depression, anxiety) with the occurrence of side effects. The only statistically significant association of a baseline measure with later reporting on the O-VRSE was that patients who reported greater hopelessness (i.e. a psychological affective process) had fewer feelings of panic when in VR. Side effects were not associated with the number of VR sessions attended or the total time spent in VR. Gains on the trial primary outcomes, agoraphobic avoidance and distress, were not associated with the occurrence of side effects. Greater feelings of value from having access to VR were associated with greater reductions in agoraphobic distress at 6 weeks (end of treatment). The O-VRSE factor scores were compared between the patients who were very satisfied (*n* = 75) and the patients who were mostly satisfied (*n* = 37). The very satisfied patients reported fewer difficulties concentrating when wearing a headset (mean = −0.26, s.d. = 0.80) than the mostly satisfied patients (mean = 0.52, s.d. = 1.53), *t*(*df* = 45.95) = −2.90, *p* = 0.006. There were no statistically significant differences between those who were very satisfied compared to those who were mostly satisfied in feelings of panic when using VR, *t*(*df* = 46.23) = −1.68, *p* = 0.099, worries following VR, *t*(*df* = 110) = −0.65, *p* = 0.520, or feeling valued for having access to VR, *t*(*df* = 110) = 1.41, *p* = 0.160.

**Table 5.** Associations of Oxford-VR side effects checklist factor scores

	Oxford-VR side effects checklist factor scores			
	Difficulties concentrating when wearing a headset	Feelings of panic using VR	Worries following VR	Feeling valued
	*R p* Value (*n*)	*R p* Value (*n*)	*r p* Value (*n*)	*r p* Value (*n*)
Baseline measures				
Agoraphobic avoidance (O-AS)	−0.05	−0.00	−0.02	−0.90
	0.572	0.981	0.819	0.323
	(117)	(117)	(117)	(117)
Agoraphobic distress (O-AS)	−0.02	0.01	0.05	0.09
	0.833	0.955	0.620	0.344
	(118)	(118)	(118)	(118)
Agoraphobia (AMI)	−0.02	0.03	0.07	−0.02
	0.828	0.716	0.453	0.819
	(114)	(114)	(114)	(114)
Paranoia – reference (R-GPTS)	−0.07	−0.03	−0.12	0.07
	0.479	0.770	0.221	0.466
	(108)	(108)	(108)	(108)
Paranoia – persecutory (R-GPTS)	−0.10	−0.11	−0.16	0.11
	0.306	0.272	0.098	0.255
	(108)	(108)	(108)	(108)
Paranoia – total (R-GPTS)	−0.09	−0.08	−0.15	0.10
	0.345	0.421	0.118	0.305
	(108)	(108)	(108)	(108)
Paranoia worries (PWQ)	−0.04	−0.08	−0.07	0.15
	0.685	0.412	0.502	0.116
	(107)	(107)	(107)	(107)
Depression (PHQ-9)	−0.06	−0.08	−0.01	−0.10
	0.511	0.431	0.922	0.306
	(112)	(112)	(112)	(112)
Threat cognitions (O-CDQ)	−0.05	0.02	0.02	−0.01
	0.579	0.855	0.862	0.881
	(116)	(116)	(116)	(116)
Avoidance (O-CDQ)	−0.08	−0.00	0.12	0.03
	0.370	0.982	0.209	0.737
	(116)	(116)	(116)	(116)
Within-situation defences (O-CDQ)	−0.04	−0.02	−0.04	0.15
	0.701	0.802	0.659	0.119
	(116)	(116)	(116)	(116)
Recovery (QPR)	0.06	−0.02	−0.09	0.18
	0.535	0.853	0.319	0.058
	(118)	(118)	(118)	(118)
Voices	0.01	−0.01	0.02	−0.01
	0.953	0.908	0.843	0.930
	(107)	(107)	(107)	(107)
Hopelessness (BHS)	−0.14	−0.21*	−0.13	−0.09
	0.142	0.028	0.177	0.387
	(107)	(107)	(107)	(107)
Therapy time				
Total number of sessions	−0.15	−0.05	−0.12	−0.06
	0.103	0.596	0.185	0.520
	(118)	(118)	(118)	(118)
Total time in VR	−0.17	−0.11	−0.08	−0.07
	0.074	0.254	0.377	0.429
	(118)	(118)	(118)	(118)
Primary outcomes				
Change in avoidance (0–6 weeks)	−0.03	−0.08	−0.08	0.09
	0.790	0.422	0.384	0.326
	(114)	(114)	(114)	(114)
Change in avoidance (0–26 weeks)	−0.09	−0.03	−0.03	0.01
	0.337	0.785	0.726	0.906
	(111)	(111)	(111)	(111)
Change in distress (0–6 weeks)	−0.07	−0.00	−0.03	0.25*
	0.490	0.991	0.721	0.008
	(115)	(115)	(115)	(115)
Change in distress (0–26 weeks)	−0.01	−0.03	−0.01	0.17
	0.882	0.733	0.934	0.074
	(111)	(111)	(111)	(111)

*p < 0.05.

## Discussion

Over the coming years VR interventions are highly likely to be provided in clinical services for patients with psychosis. Automated delivery, and new standalone headsets that do not require connection to a computer or external tracking devices, make the technology potentially implementable at scale. In this study we report for the first time on patient satisfaction with automated VR therapy supported by a mental health worker and also on the potential occurrence of side effects that could limit successful uptake of VR therapy. Almost all patients were satisfied with the VR therapy. Moreover, patients felt valued for being given access to an immersive technology that most people have not tried. This is consistent with our clinical experience, and studies of patient and staff views (e.g. Bond et al., 2021; Brown et al., 2022) that indicate substantial enthusiasm for VR approaches for mental health difficulties. Positive experiences were much more common than side effects. Importantly, side effects during the trial did not affect uptake of VR or therapeutic gains. Overall the study results indicate that patient experience will not hinder the implementation at scale of automated VR therapy but is instead more likely to prove a facilitator.

The side effects did not impact on outcomes, but it certainly should not be overlooked that patients can experience difficulties with VR. The side effects grouped into three types, which may provide a helpful framework when conceptualising the occurrence of side effects in the use of VR for the treatment of mental health conditions. Clinicians should be aware of patient fears about what is happening in the room while they are wearing a headset, that wearing a headset can induce feelings of panic, and that there may be worries that remain after a session. Difficulties concentrating when wearing a headset because of concerns about walking into something or what else may be happening in the room were more likely for patients who were mostly satisfied compared to those who were very satisfied. Understandably, side effects may bring satisfaction ratings down from the highest category. This will need addressing in implementation. For example, patients can be told of these potential side effects and time provided to discuss the best way to limit their occurrence or respond if they do occur. Clearly, the study points to the need to consider what is happening in the room while the person's view is occluded by the headset. None of the side effect sub-types were significantly predicted by individual psychiatric symptoms assessed before receiving VR therapy, such as levels of paranoia, hallucinations, or anxiety. This is an important finding for implementation; if an individual would like to try VR, services can be reassured that severity of these psychiatric symptoms is unlikely to lead to side effects.

The unpleasantness of cybersickness could be the side effect that most limits widespread adoption of VR. Three checklist items assessed feelings of sickness. Five per cent of patients reported feelings of sickness, although no one vomited. Our previous studies indicate that patients with psychosis score highly for simulator sickness even before entering VR, possibly due to affective symptoms or medication side effects, and that these symptoms do not then increase from time in VR (Fornells-Ambrojo et al., 2008; Freeman et al., 2010). We did not use the Simulator Sickness Questionnaire (Kennedy, Lane, Berbaum, & Lilienthal, 1993), the most often used assessment of cybersickness, as it was not developed for study of VR in the context of mental health difficulties. As such, almost all items overlap with symptoms of anxiety (e.g. sweating, dizziness, difficulty concentrating). In the design of gameChange we took several precautions to limit cybersickness: uninterrupted and accurate VR tracking, rapid updating of images (a sustained frame rate near 90 Hz), and minimising changes in position in VR that were not self-initiated. Nevertheless, for a small minority VR was still associated with unpleasant feelings of sickness. However, there was no indication that this was at a scale to affect adoption significantly. It would be beneficial to study side effects in future service implementation outside of a randomised controlled clinical trial. The absence of significant side effects is necessary for adoption but it will not be the only factor that determines successful implementation of a healthcare technology (Greenhalgh et al., 2017).

There are caveats to our conclusions. First, participants who took part in the current study were more likely to attend VR sessions than those who did not take part, thereby potentially skewing data collection towards more positive views. It is certainly highly plausible that participants who found VR a very negative experience would not have completed the measures, although we also note that for 15% of patients in the trial both VR provision and data collection for this study were limited simply due to pandemic restrictions. Overall uptake of VR therapy in the trial was very high and entirely consistent with the results found from the patients in the current report. Second, we developed a new assessment, designed to capture a broad range of potential side effects, and psychometric testing was limited. For example, the factor analysis did not have a confirmatory stage due to the relatively small number of participants who had VR sessions. The measure has face validity, was developed with the involvement of people with lived experience, and is easy to use, but it will require further validation as VR becomes more widely used. We view the checklist as a starting point for the study of side effects in the use of VR for mental health conditions. Third, the items were constructed for dichotomous responses, and it might be argued that this could bias responses. However, we were interested in clear occurrences of side effects, and it would not have made sense for many of the items (e.g. ‘While I was wearing the headset, I fell and injured myself.’) to be rated on Likert scales. We believe the participants understood the questions, found the scaling easy to use, and reported accurately. Importantly, a much larger participant group size is needed to ensure the stability of item parameter estimates for polytomous response formats compared to dichotomous response formats. Given the clinical nature of the research, our population size is not overly large, and therefore, using a dichotomous response format provides higher levels of stability and accuracy in the estimation of item parameters. Fourth, the positive results found for gameChange VR therapy may not always apply to other VR therapies. Different scenarios and tasks, programming quality, and hardware could all potentially produce lower levels of satisfaction and greater side effects. It is implausible that VR therapies will be equivalent in patient experience. Therefore it is important that in the future each automated VR therapy is carefully evaluated to ensure it provides a high-quality patient experience and thus can be successfully implemented at scale.

## Supplementary material

For supplementary material accompanying this paper visit https://doi.org/10.1017/S0033291722001167. click here to view supplementary material
