# Supplementary material for: Virtual reality (VR) therapy for patients with psychosis: satisfaction and side effects
Source: Psychol Med. 2022 Apr 28;53(10):4373–84. doi: 10.1017/S0033291722001167 (PMC10388321; doi:10.1017/S0033291722001167)
Supplement: Supplementary file 1 [file S0033291722001167sup001.docx]

**SUPPLEMENTARY MATERIALS**

Table S1. Factor correlations

| Factors | F1: Worries following VR | F2: Feelings of panic using VR | F3: Feeling valued | F4: Difficulties concentrating when wearing a headset |
| --- | --- | --- | --- | --- |
| F1: Worries following VR | 1.00 | 0.40 | -0.03 | 0.26 |
| F2: Feelings of panic using VR | 0.40 | 1.00 | -0.12 | 0.24 |
| F3: Feeling valued | -0.03 | -0.12 | 1.00 | -0.02 |
| F4: Difficulties concentrating when wearing a headset | 0.26 | 0.24 | -0.02 | 1.00 |
| Factor correlations are calculated based on the factor loadings (based on the oblimin rotation method). | | | | |

Table S2. Factor score correlations

| Factors scores | F1: Worries following VR | F2: Feelings of panic using VR | F3: Feeling valued | F4: Difficulties concentrating when wearing a headset |
| --- | --- | --- | --- | --- |
| F1: Worries following VR | 1.00 | 0.73 | -0.10 | 0.53 |
| F2: Feelings of panic using VR | 0.73 | 1.00 | -0.20 | 0.55 |
| F3: Feeling valued | -0.10 | -0.20 | 1.00 | -0.10 |
| F4: Difficulties concentrating when wearing a headset | 0.53 | 0.55 | -0.10 | 1.00 |
| Factor score correlations are calculated based on the factor scores of the participants (based on the least square regression method). | | | | |

**Oxford - VR Side Effects Checklist**

We would like to check whether there are any downsides to the VR therapy. Please read the below items and rate whether or not this is something that occurred for you during the course of the VR therapy. Rate each item by putting a tick under either “yes” (it did occur) or “no” (it did not occur).

| **During the course of the VR therapy:** | | **Yes** | **No** |
| --- | --- | --- | --- |
| 1. | The headset made me feel trapped/claustrophobic and I had a panic attack. |  |  |
| 2. | While I was wearing the headset, I walked into something and injured myself. |  |  |
| 3. | While I was wearing the headset, I fell and injured myself. |  |  |
| 4. | I couldn’t concentrate on my session because I was constantly thinking about what else might be happening in the room. |  |  |
| 5. | I couldn’t fully engage in the session because I was constantly thinking about crashing into something. |  |  |
| 6. | I couldn’t concentrate on my session because I was constantly thinking about what the headset might be doing to me. |  |  |
| 7. | Wearing the headset caused me pain and discomfort for quite some time after the session had finished. |  |  |
| 8. | Wearing the headset left me with worrying/distressing marks on my face for quite some time. |  |  |
| 9. | After wearing the headset, I felt so unsteady that I had difficulties walking. |  |  |
| 10. | Using the headset strained my eyes so I couldn’t see properly. |  |  |
| 11. | Using the headset gave me a lasting headache. |  |  |
| 12. | After my session, I was really concerned that the headset had messed with my thoughts. |  |  |
| 13. | After my session, I got worried and fearful about what the headset had done to me. |  |  |
| 14. | Wearing the headset made my voices worse for the rest of the day. |  |  |
| 15. | In the days after using VR, I couldn’t tell the difference between the computer VR world and the real world. |  |  |
| 16. | As a result of using VR, I was really confused about what was real and what was not real. |  |  |
| 17. | While using VR, I felt so sick that I had to stop. |  |  |
| 18. | For hours after using VR, I felt sick/unwell. |  |  |
| 19. | VR made me throw up. |  |  |
| 20. | After using VR, the everyday world felt very unreal. |  |  |
| 21. | After using VR, I felt very disconnected from the real world. |  |  |
| 22. | After using VR, I began to see disturbing things that other people couldn’t see. |  |  |
| 23. | The people in VR were so creepy that I did not want to continue with the therapy. |  |  |
| 24. | Going into the VR environments made me have panic attacks. |  |  |
| 25. | Going into the VR environments made me even more worried about other people. |  |  |
| 26. | The virtual coach was very unhelpful and put me off the therapy. |  |  |
| 27. | The therapy got too hard, too quickly, and I felt defeated. |  |  |
| 28. | Using the VR equipment made me feel really special. |  |  |
| 29. | The headset felt comfortable. |  |  |
| 30. | I felt proud of myself for being able to use the VR. |  |  |
| 31. | Receiving this new and high-tech therapy made me feel valued. |  |  |
| 32. | Using the VR equipment made me feel excited. |  |  |
| 33. | Using the VR equipment made me feel optimistic. |  |  |
